# Variations of Time Irreversibility of Heart Rate Variability Under Normobaric Hypoxic Exposure

**DOI:** 10.3389/fphys.2021.607356

**Published:** 2021-03-05

**Authors:** Yang Li, Jianqing Li, Jian Liu, Yong Xue, Zhengtao Cao, Chengyu Liu

**Affiliations:** ^1^School of Instrument Science and Engineering, Southeast University, Nanjing, China; ^2^Air Force Medical Center, Beijing, China

**Keywords:** time irreversibility, hypoxia, heart rate variability, delay time, autonomic nervous system

## Abstract

In the field of biomedicine, time irreversibility is used to describe how imbalanced and asymmetric biological signals are. As an important feature of signals, the direction of time is always ignored. To find out the variation regularity of time irreversibility of heart rate variability (HRV) in the initial stage of hypoxic exposure, the present study implemented 2 h acute normobaric hypoxic exposure on six young subjects who have no plateau or hypoxia experiences; oxygen concentration was set as 12.9%. Electrocardiogram (ECG) signals were recorded in the whole process and RR interval sequences were extracted. Mathematical operations were executed to transform the difference of adjacent RR intervals into proportion and distance with delay time to conduct time irreversibility analysis of HRV. The same calculating method was implemented on six items randomly picked out from the MIT-BIH normal sinus rhythm database as a control group. Results show that variation of time irreversibility of HRV in a hypoxic environment is different from that in a normoxic environment, time irreversibility indices of a hypoxic group decreases continually at a delay time of 1 and 2, and indices curves of time irreversibility gradually tend to be steady and gather with each other at a delay time of 3 or 4. The control group shows no consistent tendency no matter what the delay time is in the range of 1–4. Our study indicates that in short-time hypoxic exposure, as hypoxic time goes by, regulation of the cardiovascular autonomic nervous system weakens; regulation times and intensity of sympathetic and parasympathetic nerves tend to be equal.

## Introduction

Time irreversibility is one of the basic features of nonlinear systems. It describes the process of how the physical state and statistical characteristics of a system depend on the direction of time (Yao et al., 2019). If a sequence has the same joint probability distribution with its reversal sequence, it is said to be time invertible. Invertible processes include Gaussian linear processes and isentropic processes. On the contrary, time irreversibility of series indicates the existence of nonlinear processes in the underlying dynamics, including non-Gaussian stochastic processes and dissipative chaos (Lacasa et al., 2012). The heartbeat of normal individuals is jointly controlled by multiple factors of sympathetic and parasympathetic nerves, and thus the time series of cardiac intervals shows characteristics of chaos, nonlinearity, and complexity (Rajbhandari Panday and Panday, 2018). Costa et al. (2005) pointed out that time irreversibility of cardiac intervals is influenced by age and pathological factors; healthy young people have significantly higher time irreversibility of cardiac intervals than that of healthy elderly people, and healthy aged people have distinctly higher time irreversibility of cardiac intervals than patients with congestive heart failure (CHF) or atrial fibrillation. This conclusion was also supported by Hou et al. (2011) with multi-scale time irreversibility method, and she also indicated that there are significant differences of time irreversibility in healthy young people between day and night (Hou et al., 2012).

Many methods have been proposed for the measurement of time irreversibility of cardiac interval sequences. Differentiate operations between adjacent RR intervals is used by Porta et al. (2006) who took the occurrence frequency of adjacent intervals’ difference greater than or less than zero as the time irreversibility index. And Costa et al. (2008) introduced the coarse-grained multi-scale method on Porta’s work. Guzik et al. (2006) performed scaling operations on the square of RR intervals. Lacasa et al. (2012) traced the amplitude of each point in the sequence with a graph, calculated the number of points that each point could reach without crossing with other points, and constructed a new sequence as the calculating basis of time irreversibility. Ordinal mode has been applied to calculate the emergence frequency of various patterns (Graff et al., 2013). Hou et al. (2011) defined high-dimensional irreversibility calculation index on the work of Porta (Graff et al., 2013) and Guzik (Guzik et al., 2006) by introducing different delay times for irreversibility analysis, and the mean value of all calculation results is the value of high-dimensional irreversibility. It reflects the comprehensive influence of different levels’ time delay of nerves and fluid in the human body on time irreversibility of heart rate variability (HRV). And research shows that both age and exercise have a great effect on high-dimensional irreversibility (Hou et al., 2011, 2013).

Hypoxia is one of the stimulating factors that make HRV change. When entering a hypoxic environment, blood oxygen saturation (SpO_2_) of the human body decreases, and regulation of the autonomic nervous system changes, which is usually manifested as an accelerated heart rate and shortened breath (Xue et al., 2010; Liu et al., 2019). Lower oxygen concentration may even result in altitude sickness, with symptoms such as headache and nausea (Zhang et al., 2014). Different methods have been applied to assess how HRV changes when people are exposed to a hypoxic environment. Most of the research results found that the standard deviation (SDNN) and root mean square (RMSSD) between adjacent heartbeat intervals and low frequency (LF) and high frequency (HF) in the spectrum decrease while the ratio of LF to HF increases (Krejčí et al., 2018) and sample entropy declines (Zhang et al., 2014) at the same time. However, other researchers gave different results (Iwasaki et al., 2006; Vigo et al., 2010). Normobaric hypoxic chambers are often used in laboratories to simulate the plateau hypoxic environment. It creates hypoxic effects by filling the chamber with excess nitrogen to dilute the air, which is safe, economic, and easy to use.

Existing studies mostly compare the physiological state under normoxia and hypoxia and find out how physiological indicators change after people are exposed to a hypoxic environment. For example, variation of electrocardiogram (ECG) in hypoxic exposure was studied in Coustet et al. (2015); variation tendencies of HRV and SPO_2_ within 10 min of acute hypoxic exposure were revealed in Krejčí et al. (2018). Some other studies explored people’s hypoxic acclimatization speed or physical training methods through experiments (Beidleman et al., 2008; Zeng et al., 2013, 2019; Sinex and Chapman, 2015). As one of the methods to evaluate the health of the physiological system, time irreversibility analysis method has been widely used in medical research. It can reflect the pathological information of the cardiovascular system and aging status, however, this method has never been used in the study of human physiological variation in a hypoxic environment. Some symptoms that occur in hypoxia are similar to other diseases and there must be connections between time irreversibility and those symptoms. We need to conduct research over a continuous period of time at the beginning of hypoxia to study the subtle variation trends of physiological indicators. This research is of great importance for the prevention of acute altitude sickness, the physical training of pilots and astronauts in response to accidents of loss of pressure, and the revelation of dynamics and autonomic rhythm changes of the human cardiovascular nervous system in the early stage of hypoxic exposure. In the process of cardiac feedback control, delay happens at all levels of the physiological system due to the reaction speed of chemical transmitters (Casali et al., 2008; Alvarez-Ramirez et al., 2009), and some useful information that the data can express may be lost if time irreversibility is calculated only based on the difference between adjacent RR intervals. In order to explore variation laws of time irreversibility of HRV in the early stage of hypoxic exposure, experiments will be carried out in a normobaric hypoxic chamber. By introducing different delay times, time irreversibility variation rules of HRV will be studied in an acute 2 h hypoxic exposure. Few reports have been posted on the research of time irreversibility of HRV in a hypoxic environment.

## Materials and Methods

### Subjects

Six young and healthy male volunteers were recruited for our experiment. Over 24 h stay at an altitude >3,000 m in the previous 6 months, born at an altitude >1,000 m, being a smoker, or having a history of severe respiratory or cardiopulmonary diseases are the exclusion criteria. All subjects were forbidden to consume tea, coffee, alcohol, medicine that could get the neural system excited, or do intense physical exercise 24 h prior to the experiment. Characteristics of the subjects are presented in Table 1. The study was approved by the Ethics Committee of Southeast University, China. All the subjects were well informed of the aim and risks of the experiment and they all signed their informed consent before the study.

**TABLE 1:** Characteristics of subjects.

Body mass (kg)	65.8 ± 6.0
Body height (cm)	173.3 ± 5.1
Body mass index (kg/m^2^)	21.9 ± 1.3
Age (years old)	20.4 ± 1.5
Smoker	None
Prior altitude >3,000 m experience	None
History of severe respiratory or cardiopulmonary disease	None

*Data are listed as means ± SD or as number.*

### Experimental Design and Data Acquisition

This experiment was implemented in a normobaric hypoxic chamber. Before the experiment, nitrogen was filled into the chamber to reduce the oxygen concentration to 12.9% (equivalent to the altitude of 3,600 m), then subjects entered the chamber. During the experiment, the nitrogen flow was controlled by an automatic valve (Yu et al., 2011), so that the oxygen concentration in the chamber was maintained at 12.9% ± 0.2%. The experiment lasted for 2 h, and subjects were asked to keep quiet and lie in their bunks. They could watch cellphones, read books, and listen to gentle music. ECG signals were recorded by tiny equipment inlaid in a chest belt for each subject (SensEcho-5A, Health Regulation Co. Ltd., Beijing, China). The sampling rate was 200 Hz, and digitized ECG signals were stored in a built-in hard disk, which is capable of storing 24 h of data. The temperature and humidity in the chamber were maintained at 22 ± 1°C and 25 ± 1%, respectively.

### Data Processing and Calculating Method

First, we identified each QRS complex automatically by P&T methods (Pan and Tompkins, 1985) for its extensively tested accuracy and efficiency. False and missing detection were calibrated artificially. Then we extracted R-R intervals series by corrected R wave positions.

Porta et al. (2006) counted the number of times that the difference of one RR interval with the next was less than zero and when the number was unequal to zero; the two numbers’ ratio was defined as time irreversibility index *P*%. Guzik et al. (2006) performed arithmetical operations on the square of the difference between adjacent RR intervals and defined time irreversibility index *G*%. We denote any element in RR intervals sequence as *RR*_*i*_(1≤*i*≤*n*), where *n* is length of RR intervals sequence. The difference between adjacent RR intervals is shown as Δ*RR* = *RR*_*i*_−*RR*_*i*−1_. Δ*RR* is also expressed as Δ*RR*^+^ when Δ*RR* > 0, and Δ*RR*^−^ when Δ*RR* < 0. *N*(Δ*RR*^+^) is the number of Δ*RR*^+^ in the entire Δ*RR* sequence. Delay time τ is introduced in this study. For τ = 1,2,…,*n*−1, Δ*RR*_τ_ = *x*_*i* + τ_−*x*_*i*_(1≤*i*≤*n*−τ), time irreversibility indices *P*%(τ) and *G*%(τ) for each τ are given by

(1) $$P\%\,\left(\tau \right)=\frac{N\,\left(\Delta \,R\,R_{\tau }^{-}\right)}{N\,\left(\Delta \,R\,R_{\tau }^{-}\right)+N\,\left(\Delta \,R\,R_{\tau }^{+}\right)}\times 100,$$

(2) $$G\%\,\left(\tau \right)=\frac{\sum_{i=1}^{N\,\left(\Delta \,R\,R_{\tau }^{+}\right)}\Delta \,R\,R_{\tau }^{+}\,{\left(i\right)}^{2}}{\sum_{i=1}^{N\,\left(\Delta \,R\,R_{\tau }^{+}\right)}\Delta \,R\,R_{\tau }^{+}\,{\left(i\right)}^{2}+\sum_{i=1}^{N\,\left(\Delta \,R\,R_{\tau }^{-}\right)}\Delta \,R\,R_{\tau }^{-}\,{\left(i\right)}^{2}}\times 100.$$

Distance between *P*%(τ), *G*%(τ) and 50 are expressed as

(3) $$Q\,P_{\tau }=\left|50-P\%\,\left(\tau \right)\right|,$$

(4) $$Q\,G_{\tau }=\left|50-G\%\,\left(\tau \right)\right|.$$

In order to make comparisons with the hypoxic group, we randomly selected six items of 2 h from the MIT-BIH Normal Sinus Rhythm Database (Goldberger et al., 2000) to perform the same time irreversibility analysis on ECG data. We will take *QP*_τ_ and *QG*_τ_ as the measurement of time irreversibility.

## Results

Since *QP*_τ_ and *QG*_τ_ are all statistics independent of the sequence length, we calculated each index value from the beginning of the experiment and moved forward by 1 min each time. The first computation scope is [0,5 min]. That is, we calculated the indexes in the scope of [0,5 min], [0,6 min], [0,7 min], …, [0,120 min], respectively, and drew line graphs. In this way, data volume for calculation is gradually increased and the curve is relatively flat. Interference for observation and analysis because of a short or sharp change in a certain data section can be avoided.

### Variation of *QP*_1_, *QG*_1_, *QP*_2_, and *QG*_2_

**FIGURE 1:**
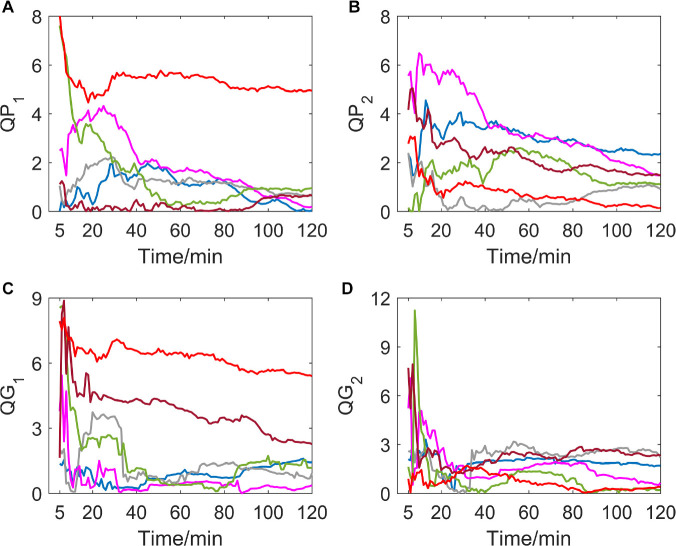
**(A–D)** show the corresponding results of *QP*_1_, *QP*_2_, *QG*_1_, and *QG*_2_. Variation tendency of *QP*_1_, *QP*_2_, *QG*_1_, and *QG*_2_ within 2 h of hypoxic group (each color represents a subject, the same below).

If delay time was set to be 1 or 2 (as shown in Figure 1), indexes of all subjects in the hypoxic group fluctuated greatly in the first 15 min after the experiment began, which is possibly due to the data volume for calculation being small meaning the autonomic nervous system of the human body must undergo the process of adjustment and adaptation when first entering the hypoxic environment. In the entire experiment, the trends of *QP*_1_, *QG*_1_, *QP*_2_, and *QG*_2_ are generally toward zero, which indicates time irreversibility of HRV of the hypoxic group tends to decline.

**FIGURE 2:**
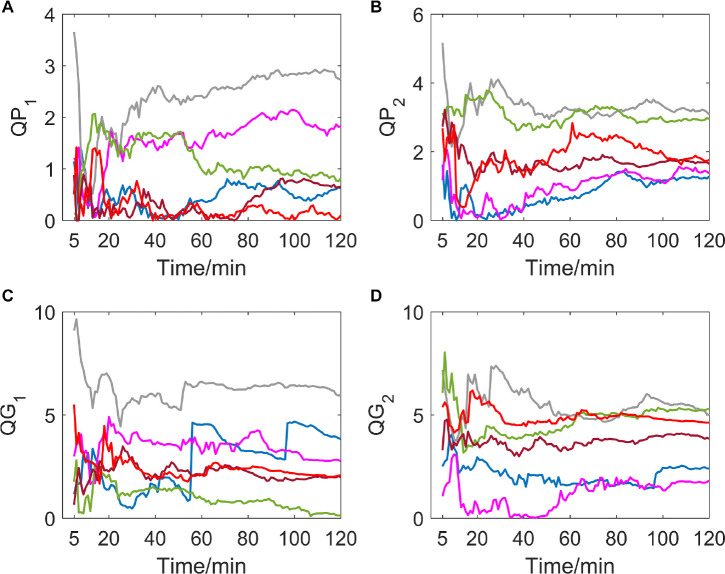
**(A–D)** show the corresponding results of *QP*_1_, *QP*_2_, *QG*_1_, and *QG*_2_. Variation tendency of *QP*_1_, *QP*_2_, *QG*_1_, and *QG*_2_ within 2 h of MIT normoxic group.

Different from the hypoxic group, which showed a significant downward trend of *QP*_1_, *QG*_1_, *QP*_2_, and *QG*_2_, indicators of the MIT normoxic group did not show an obvious consistent changing direction (as shown in Figure 2).

**FIGURE 3:**
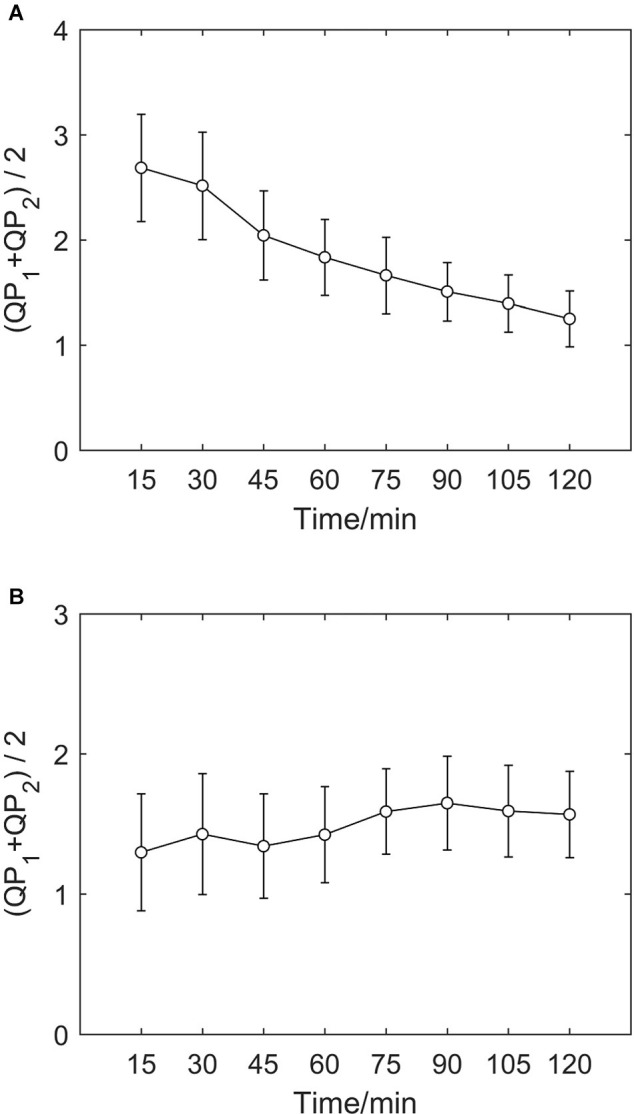
Average value error bar chart of *QP*_1_ and *QP*_2_ of two groups. **(A)** Error bar chart of hypoxic group. **(B)** Error bar chart of MIT normoxic group.

If we calculate indicators every 15 min and take both τ = 1 and τ = 2 into consideration, we can draw an error bar chart with the average value of *QP*_1_ and *QP*_2_ (as shown in Figure 3), which clearly shows the average time irreversibility of all subjects with a delay time of 1 and 2 in different groups. As in the charts above, indicators of the hypoxic group decreases and the MIT normoxic group shows no clear differences.

### Variation of *QP*_3_, *QG*_3_, *QP*_4_, and *QG*_4_

**FIGURE 4:**
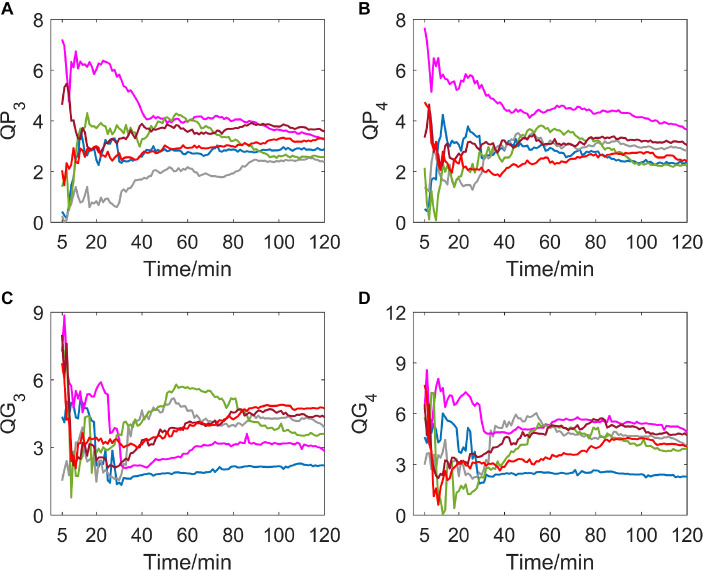
**(A–D)** show the corresponding results of *QP*_3_, *QP*_4_, *QG*_3_, and *QG*_4_. Variation tendency of *QP*_3_, *QP*_4_, *QG*_3_, and *QG*_4_ within 2 h of hypoxic group.

**FIGURE 5:**
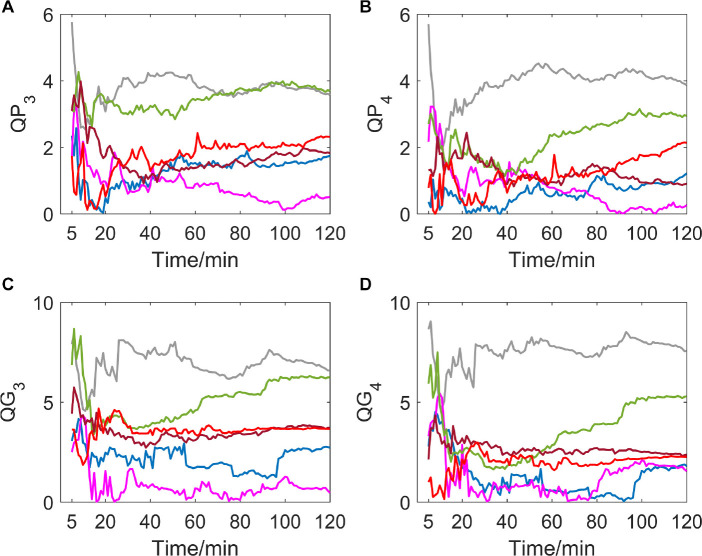
**(A–D)** show the corresponding results of *QP*_3_, *QP*_4_, *QG*_3_, and *QG*_4_. Variation tendency of *QP*_3_, *QP*_4_, *QG*_3_, and *QG*_4_ within 2 h of MIT normoxic group.

If delay time was set to be 3 or 4 (as shown in Figure 4), a great difference in variation trend happened in *QP* and *QG* from that when delay time is 1 or 2. Indicators no longer show a slow downward trend toward zero with time going by, but gradually tend to be stable after the drastic transition period of the first 30 min. And most of the subjects’ indicator curves gradually converge to a narrow range. In contrast, there was no significant trend change in the MIT normoxic group (as shown in Figure 5).

## Discussion

Research on time irreversibility of HRV in a hypoxic environment has not been carried out before. Previous studies have shown that age and pathology will lead to a decrease of time irreversibility of HRV (Costa et al., 2005) and, compared to the daytime, time irreversibility of both healthy people and patients with congestive heart failure (CHF) declines at night (Porta et al., 2009). Our study in a hypoxic environment reveals a similar rule: prolonged hypoxic exposure leads to a sustained decline of time irreversible of HRV within 2 h when the delay time is 1 or 2.

Hypoxia is one of induction factors that can make regulatory mechanisms of cardiovascular nervous system change: parasympathetic activity was significantly reduced and autonomic nervous system regulation was greatly inhibited (Zhu et al., 2010). Since healthy physiological systems have the strongest nonlinearity and complexity, the decrease of time irreversibility in an anoxic state indicates a depression of nonlinearity and complexity.

In RR interval series, the positive difference between adjacent RR intervals represents a sudden increase of heart rate due to sympathetic modulation, whereas the negative difference represents a decrease in instantaneous heart rate due to parasympathetic modulation. The indicators variation of an MIT normoxic group reveals that there is always a certain difference of regulation times between sympathetic and parasympathetic nerves in normal individuals under normal oxygen concentration, while the difference tends to diminish in hypoxic environments. By definition, *QP*_1_ reflects the absolute difference of regulation times between sympathetic and parasympathetic nerves, and *QG*_1_ is a measurement of regulation intensity by calculating the square of RR intervals difference. The fact that both of them are approaching zero indicates that regulation times and intensity of sympathetic and parasympathetic nerves tend to be close to each other.

The LancetIn the present study, we observed a decrease in time irreversibility of the hypoxic group when the delay time was 1 and 2, but the variation trend changed when the delay time was increased to 3 or 4. It can be seen that the trend of *QP*_3_, *QG*_3_, *QP*_4_, and *QG*_4_ in the hypoxic group gradually becomes stable and converges with each other, while there is no significant difference whatever the delay time is of the MIT normoxic group, which indicates that time irreversibility of HRV is closely related to the delay time in a hypoxic environment. The results of mutual information analysis shows that the delay time of RR interval sequence in the elderly was significantly longer than that in the young (Ma and Zhang, 2010). Further research is needed to find out what generates the difference of delay time between hypoxic and normoxic environments. Hou et al. (2011) defined the average value at different delay times of *QP* and *QG* as the high dimensional time irreversibility index *P*_*m*_, *G*_*m*_, and the square root of their sum of squares was defined as their comprehensive measurement index *D*_*m*_. Her study pointed out that the relationship between age, CHF, and time irreversibility of HRV was affected by the embedded dimension. When the value range of the embedded dimension changed, the variation rule revealed by time irreversibility indicators also changed. If the embedding dimension is *m*, the essential meaning of *P*_*m*_ and *G*_*m*_ is the average value of *QP* and *QG* when the delay time is taken from 1 to *m*. In the multi-level control loop and feedback network system of the human body, they can reflect the average effect of signals derived from different levels and different delays, but researchers cannot make observations on the specific value of time irreversibility index under a certain delay time. When the value of delay time increases continuously, variation of *P*_*m*_ and *G*_*m*_ must become gentler and have the characteristics of lag. It was found that time irreversibility of HRV during exercise was significantly increased when taking multiple dimensions into consideration (Hou et al., 2013). Time irreversibility of HRV in a short period of time at the beginning of hypoxic exposure was investigated in this paper. Studies have shown that intermittent hypoxic training over several days significantly improves SDNN, RMSSD, LF HF (Xu et al., 2004), and SPO_2_ (Fulco et al., 2013), however, the impact on time irreversibility needs further research.

## Conclusion

Variation laws of time irreversibility of HRV in a normobaric hypoxic environment were researched in the present study. We found that in 2 h hypoxic exposure, time irreversibility indicators *QP*_1_, *QG*_1_, *QP*_2_, and *QG*_2_ decrease continuously, while *QP*_3_, *QG*_3_, *QP*_4_, and *QG*_4_ gradually tend to be stable and converge to a narrow range, which means there is a relationship between time irreversibility of HRV and delay time, and that proper selection of delay time is important for the observation of the relationship. In contrast, normoxic environments do not have any influence on time irreversibility of HRV whatever the delay time is. Results when delay time is 1 or 2 suggests a decline in automatic nervous system activities in a hypoxic environment, and regulation times and intensity of sympathetic and parasympathetic nerves get close to each other.

## Data Availability Statement

The raw data supporting the conclusions of this article will be made available by the authors, without undue reservation.

## Ethics Statement

The studies involving human participants were reviewed and approved by the Ethics Committee of Southeast University, Nanjing, China.

## Author Contributions

YL did all the analysis work and wrote the article. JqL checked research protocol. JL performed the data calibration. YX conducted the experiment and collected data. ZC provided the experimental site and equipment. CL guided the research method, wrote the article and examined the research results. All authors contributed to the article and approved the submitted version.

## Conflict of Interest

The authors declare that the research was conducted in the absence of any commercial or financial relationships that could be construed as a potential conflict of interest.
